# Detection of Unamplified *E. coli* O157 DNA Extracted from Large Food Samples Using a Gold Nanoparticle Colorimetric Biosensor

**DOI:** 10.3390/bios12050274

**Published:** 2022-04-26

**Authors:** Emma Dester, Kaily Kao, Evangelyn C. Alocilja

**Affiliations:** 1 Nano-Biosensors Lab, Department of Biosystems and Agricultural Engineering, Michigan State University, East Lansing, MI 48824, USA; desterem@msu.edu (E.D.); kaokaily@msu.edu (K.K.); 2 Global Alliance for Rapid Diagnostics, Michigan State University, East Lansing, MI 48824, USA

**Keywords:** foodborne pathogens, foodborne illness, biosensing, food safety, oligonucleotide probe

## Abstract

Rapid detection of foodborne pathogens such as *E. coli* O157 is essential in reducing the prevalence of foodborne illness and subsequent complications. Due to their unique colorimetric properties, gold nanoparticles (GNPs) can be applied in biosensor development for affordability and accessibility. In this work, a GNP biosensor was designed for visual differentiation between target (*E. coli* O157:H7) and non-target DNA samples. Results of DNA extracted from pure cultures indicate high specificity and sensitivity to as little as 2.5 ng/µL *E. coli* O157 DNA. Further, the biosensor successfully identified DNA extracted from flour contaminated with *E. coli* O157, with no false positives for flour contaminated with non-target bacteria. After genomic extraction, this assay can be performed in as little as 30 min. In addition, food sample testing was successful at detecting approximately 10^3^ CFU/mL of *E. coli* O157 magnetically extracted from flour after only a 4 h incubation step. As a proof of concept, these results demonstrate the capabilities of this GNP biosensor for low-cost and rapid foodborne pathogen detection.

## 1. Introduction

Each year, foodborne illnesses are responsible for hundreds of millions of cases around the world, with many ending in hospitalization or death [1]. Some of the most implicated bacterial species include *Salmonella* spp., *Campylobacter*, and Shiga toxin-producing *Escherichia coli* (STEC) [2]. In the United States alone, STEC infections were responsible for thousands of illnesses and 660 hospitalizations in 2019 [3]. STEC outbreaks can occur in a variety of food matrices, including meat products, raw flour, and leafy green vegetables [2]. Thus, rapid detection of foods contaminated with STEC is essential to protect the health and safety of all consumers.

However, many obstacles still exist for rapid and accessible detection of foodborne pathogens. Traditional enumerative techniques typically require days of sample culturing before detection; thus, rapid methods have been developed to protect consumers more effectively [4,5]. Widely implemented polymerase chain reaction (PCR) techniques, for instance, have reduced detection time to only hours [6]. Despite their advantages, PCR assays also require costly reagents, advanced laboratory equipment, and trained personnel that reduce accessibility in many low-income and middle-income countries [7,8,9]. Immunological assays such as ELISA, which rely on antibody–antigen reactions, also require highly qualified personnel as well as antibodies that increase cost and storage needs [5]. Therefore, low-cost, accessible, and rapid techniques are still necessary to address the global need for rapid foodborne pathogen detection. Many biosensors have been developed to address this need, with recent examples for *E. coli* summarized in Table 1.

As shown in the table, biosensors often are capable of rapid *E. coli* detection with limited sample preparation steps or required expertise [11,12,13,15]. However, many of these techniques (impedimetric assays, SPR, and SERS) require expensive equipment such as spectropolarimeters and spectrometers [11,12,14,15]. Additionally, other techniques require antibodies for detection, which leads to increased storage conditions and costs [10,12,13,15]. Finally, many biosensors listed were tested with bacteria from pure cultures or water samples [10,11,12]; thus, the effect of a complex food matrix is yet unknown.

Colorimetric gold nanoparticle (GNP) biosensors are one potential solution. Nanoparticle properties, including a high surface area to volume ratio, make them widely applicable in analyte capture and sensing applications [16]. GNPs in particular are easily modified with biomolecules and are chemically stable, which are key advantages for biosensor applications [16,17]. They also feature unique optical properties. The coherent oscillation of free electrons in colloidal GNP solutions produces a strong SPR (Surface Plasmon Resonance) band [18]. As this SPR band is distance dependent, aggregation of the nanoparticles leads to a visible color change [17]. Small and dispersed gold nanoparticles will feature a peak absorbance around 520 nm and appear red in color, while the aggregation of particles will lead to higher peak wavelength absorbance (approximately 600 or higher) and a visible color change to blue or purple [19]. As a result of these properties, GNPs are utilized in a variety of biosensing techniques, including piezoelectric biosensors [20], fluorescence sensing [17], optical biosensors [16,21], and electrochemical techniques [20,22]. Notably, the visible GNP color change allows for detection without expensive analytical equipment through colorimetric biosensors [23].

**Figure 1:**
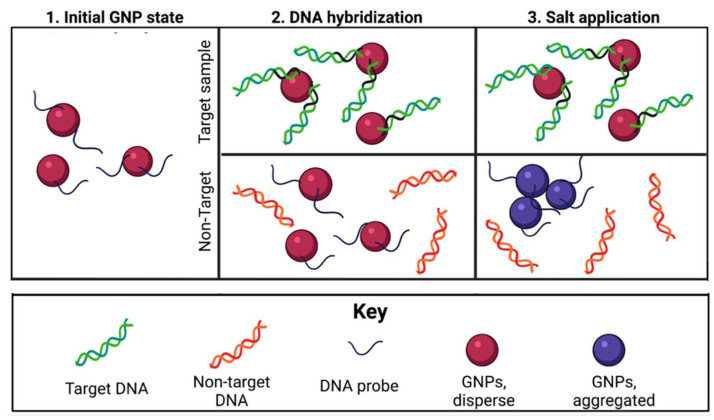
General mechanism for target-aggregating GNP colorimetric DNA biosensors.

GNP colorimetric biosensors rely on the visible color change of a solution due to the aggregation of GNPs. Methods with non-target aggregation typically use one probe sequence attached to GNPs that will bind to target DNA [24]. After DNA hybridization has occurred, a salt is added to the solution. GNPs are typically coated with adsorbed negative ions such as citrate or dextrin, whose electrostatic repulsion prevents particle aggregation; however, introduction of a salt to the colloid GNPs is known to disrupt these forces and induce particle aggregation [18,25,26]. While GNP–probe complexes bound to target DNA are protected from aggregation and remain red in color, samples without target DNA will aggregate and turn purple or blue [23,27]. This generalized mechanism is outlined in Figure 1.

Colorimetric GNP biosensors have been implemented for detection of a variety of targets, including enzymes [28,29], ions [30,31,32], and viral DNA [24]. For food pathogen detection, select biosensors have been successfully developed. For instance, one biosensor could detect 9 pg/µL of *Klebsiella* *pneumoniae* in under an hour [33]. Similarly, another biosensor detected 9.4 ng/µL of uropathogenic *E. coli* strains from pure culture in only 30 min [23]. GNP biosensor experiments have also been conducted in food matrices, with one author detecting 10 CFU/g of *Salmonella* spp. in blueberry and chicken samples after pre-treatment with IMS (immunomagnetic separation) and a 6 h sample incubation [27].

GNP colorimetric DNA biosensors feature several key advantages. For instance, the biosensors are more accessible than many other rapid biosensors due to their cost-effectiveness and small size [19,24]. In addition, as results are visually detectable, the presence of foodborne pathogens can potentially be determined without the need for expensive analytical equipment. They also rival other rapid detection methods in terms of assay duration, with DNA-based detection usually carried out in under one hour [19,23]. However, colorimetric GNP biosensors still face some challenges for foodborne pathogen detection. For instance, limited studies have been conducted on the application of these biosensors for pathogen detection directly from foods, and pre-treatment culturing steps of at least 6 h may still be required [27,33]. In addition, existing methods require days for GNP functionalization with the oligonucleotide probe, increasing the required labor for this assay [23,27]. For real-world applications, a biosensor must be developed with rapid probe functionalization and successful detection of pathogens extracted from food samples.

In this study, a gold nanoparticle-based colorimetric test with rapid DNA probe functionalization was designed for detection of *E. coli* O157:H7 extracted from food matrices. Colorimetric results were quantified through absorbance spectra measured using the NanoDrop One UV–Vis spectrophotometer. The novelties of this study are in the following areas: use of a long oligonucleotide probe (30 mers) targeting the Stx1 gene, simple probe functionalization, and direct detection of genomic unamplified target DNA extracted from artificially inoculated food samples.

## 2. Materials and Methods

### 2.1. Materials

Frozen bacterial stock cultures of *Escherichia coli* O157, *Salmonella enterica* serovar Enteritidis, and *Bacillus cereus* were obtained from the Nano-Biosensors Laboratory at Michigan State University (MSU). *Listeria monocytogenes* EGD-e was obtained from Dr. Bergholz’s Laboratory at MSU, and *Escherichia coli* C-3000 (15597) was obtained from the American Type Culture Collection (ATCC). The Powerlyzer Microbial Kit and AE buffer solution used for DNA extraction were purchased from Qiagen (Germantown, MD, USA). A NanoDrop One from ThermoFisher Scientific was used to quantify DNA concentrations and absorbance spectra data (Waltham, MA, USA). The device has a working spectral range of 190–850 nm and wavelength accuracy of ±1 nm, with full specifications detailed online and in the user manual [34,35].

Proprietary chitosan-functionalized magnetic nanoparticles (200 nm in diameter) and were used as received from the Nano-Biosensors Lab, MSU. Whirlpak bags (92 oz. and 18 oz.) were purchased from VWR International (Radnor, PA, USA). Fleximag Separators were purchased from Spherotech Inc (Lake Forest, IL, USA). Tryptic Soy Agar (TSA), Tryptic Soy Broth (TSB), Hydrochloric acid (ACS reagent, 37%), gold (III) chloride trihydrate (HAuCl_4_), sodium carbonate (Na_2_CO_3_), 11-mercaptoundecanoic acid (MUDA, HS(CH_2_)_10_CO_2_H), sodium dodecyl sulfate (SDS, C_12_H_25_NaO_4_S), and dextrin from potato starch (C_6_H_12_O_6_) were purchased from Sigma Aldrich (St. Louis, MO, USA). Phosphate Buffer Solution (PBS), pH 7.4, was prepared as directed by the supplier, Sigma Aldrich.

### 2.2. Probe Design and PCR Confirmation

The oligonucleotide probe was designed to specifically target *E. coli* O157, with a genome size of approximately 5.5 Mb [36]. The probe specifically targeted the Shiga toxin Stx1 subunit A (StxA1) gene with the following sequence: TCT GCC GGA CAC ATA GAA GGA AAC TCA. The probe was designed using NCBI BLAST (National Center for Biotechnology Information Basic Location Alignment Search Tool) and purchased with 5′ amination and a poly-A tail from Integrated DNA Technologies (Coralville, IA, USA).

Targeting the same gene, *E. coli* O157 primers (Stx1F934 and Stx1R1042) recommended by the Bacteriological Analytical Manual (BAM) [37] were also purchased from Integrated DNA Technologies. For confirmation of biosensor results, PCR was conducted on pure *E. coli* O157 DNA samples and samples extracted from flour using the Qiagen Powerlyzer kit. The PCR protocol and gel electrophoresis was adapted from existing protocols amplifying Stx genes [38].

### 2.3. GNP Synthesis and Surface Coating

GNPs were synthesized according to the procedure by Yrad et al. [39]. Briefly, dextrin-coated gold nanoparticles were synthesized using 5 mL of 2 mM gold (III) chloride trihydrate (HAuCl_4_), sterile water, 0.5 mL of 10% sodium carbonate (Na_2_CO_3_) solution, and 20 mL of dextrin. The GNPs were thiol-coated using 25 µM MUDA and resuspended in 500 µL borate buffer.

### 2.4. Bacterial Culture

Frozen stock cultures of each bacterial species were stored at −80 °C. Master plates were created by streaking 10 µL of a stock culture on TSA and incubating at 37 °C for 24–48 h. These plates were stored at 4 °C for a maximum of six weeks before replacement. Fresh bacterial cultures, “overnight transfers,” were created for each experiment by transferring a single colony from the master plate into 9 mL TSB. Transfers were incubated overnight before use.

### 2.5. Biosensor Design and Optimization

**Figure 2:**
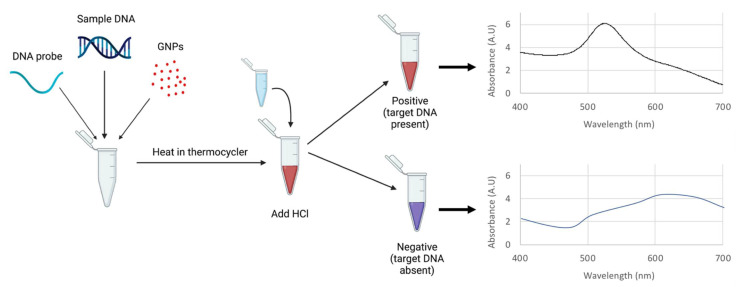
Basic procedure for GNP biosensor.

For each sample, 5 µL DNA probe, 10 µL sample DNA, and 5 µL GNPs were combined in a single tube. Samples were then heated in the thermocycler for probe hybridization. Tubes were subjected to 5 min at 95 °C (denaturing) and 10 min at 55 °C (annealing) before cooling to room temperature. This heating cycle causes target DNA (if present) to hybridize to the probe. After the tubes cooled, HCl was added. Application of salts such as HCl typically causes GNP aggregation; however, target DNA bound to the GNP-probe prevents this. Thus, samples with non-target DNA aggregated and turned purple/blue, while samples with target DNA remained red. This was quantified with absorbance spectra; red samples retained a peak wavelength close to 520 nm, while purple/blue samples shifted to higher peak wavelengths. This basic procedure is outlined in Figure 2.

Optimization variables included the amount of HCl added and the time between HCl addition and reading colorimetric results (5–15 min). The optimal HCl amount and aggregation time were determined through quantitative and qualitative analysis. First, HCl volume was optimized by adding 5 µL 0.1 M HCl at a time to negative control (water) and target (10 ng/µL *E. coli* O157 DNA) tubes at 1 min intervals until aggregation of the control without aggregation of the target tube was visually observable. The lowest HCl volume with visible control tube aggregation was then used to compare target samples to multiple non-targets, all at 10 ng/µL. Absorbance spectra readings were taken at 5 min intervals after HCl application until visible aggregation of the target samples occurred. Thus, the optimized procedure had the greatest and most consistent peak shift difference between target and non-target samples, along with a visibly red target sample when compared to the non-target and control.

### 2.6. Sensitivity and Specificity Testing

A series of 9 trials using a DNA concentration of 10 ng/µL was conducted to determine biosensor specificity. Four non-target bacterial species were represented: *Escherichia coli* C-3000, *S.* Enteritidis, *Listeria monocytogenes*, and *Bacillus cereus*. A negative control containing water and no DNA was also included. Genomic DNA extraction was achieved using the Qiagen Powerlyzer DNA extraction kit on overnight bacterial transfers. Extracted DNA was measured using Nanodrop dsDNA measurements and diluted in AE buffer to 10 ng/µL. Ten minutes after HCl application, absorbance measurements and images were collected. Results were analyzed through quantification of “peak wavelength,” the wavelength corresponding to peak absorbance.

A separate series of 9 trials was conducted to determine biosensor sensitivity. DNA collected and quantified as previously described was serially diluted to lower concentrations, ranging from 20 to 1.25 ng/µL. For each replicate, a target DNA sample was compared to a non-target sample of the same concentration. The difference in peak wavelength between target and non-target samples was then calculated at each DNA concentration level. Statistical analysis of this peak wavelength difference was then used to determine sensitivity, where a peak wavelength difference between non-target and target samples significantly greater than zero (α = 0.05) indicated sensitivity at this DNA concentration.

### 2.7. Biosensing from Large Food Samples

Bacteria extracted from food using a magnetic nanoparticle (MNP)-based extraction procedure was also tested in the biosensor. The extraction procedure was adapted to follow BAM (Bacteriological Analytical Manual) protocols, beginning with artificial contamination. Extractions were conducted in triplicate for *E. coli* O157 (T), *L. monocytogenes* (NT1), and *E. coli* C-3000 (NT2). In addition, a set of trials was conducted without artificial inoculation (NT3).

To contaminate samples, 1 mL of an overnight transfer was first added to 9 mL of TSB and incubated for 4 h. Then, 25 g of flour was weighed into a 92 oz. Whirlpak bag. Next, 1 mL of the 4 h bacterial culture was serially diluted to approximately 10^5^ CFU/mL and added to the sample. This culture was further diluted and plated on TSA to confirm the initial concentration. After artificial contamination of the flour samples, bacteria were allowed to acclimate for 1 h at room temperature before 225 mL of PBS was added to each sample. Bags were then placed in a stomacher for 2 min, and the liquified food matrix was separated into Whirlpak bags with 100 mL of liquified food each. Then, 1 mL of MNPs were added to the bag, mixed, and allowed to incubate at room temperature. After 5 min, the Whirlpak bag was attached to a magnetic rack for another 5 min before supernatant removal. The remaining sample was resuspended in 1 mL PBS.

For each concentrated sample, 500 µL was then transferred to 4.5 mL of TSB and incubated for 4 h. DNA extraction was then performed using the Qiagen Powerlyzer kit and quantified using the NanoDrop. Samples with the same bacterial inoculation were pooled for testing. DNA extracted from target-inoculated flour was then compared to two nontarget-inoculated DNA samples, as well as DNA from uncontaminated flour. Pooled DNA samples extracted from flour were compared at initial extraction concentrations. If initial concentrations between samples differed by >5 ng/µL, samples were diluted to the lowest concentration in the sample set for standardization and tested again.

### 2.8. Statistical Analysis

Statistical analysis utilized 95% confidence intervals of the wavelength corresponding to peak absorbance to compare target and non-target results. In addition, comparison of multiple groups for specificity and food testing was accomplished through the Kruskal–Wallis test and the non-parametric Student–Newman–Keuls test [40]. Sensitivity testing relied on 95% confidence intervals using the Student’s t distribution. Groups were tested with 9 replicates per sample (*n* = 9) for all pure cultures and 6 replicates per sample (*n* = 6) for studies in samples extracted from flour.

## 3. Results and Discussion

### 3.1. Principles of the E. coli O157 Nano-Biosensor

**Figure 3:**
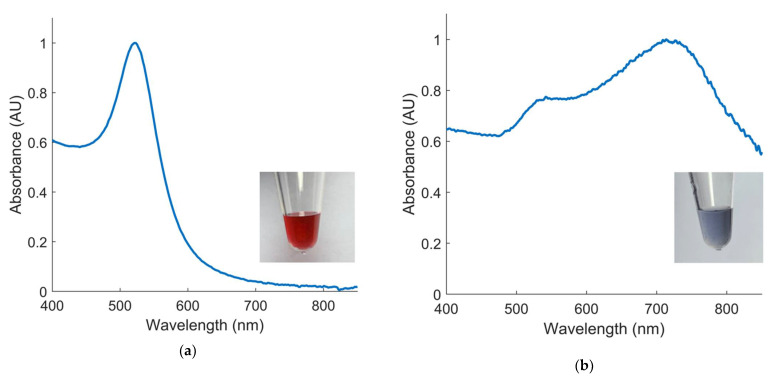
Normalized absorbance spectra and visual appearance of (**a**) pure colloidal GNPs and (**b**) GNPs aggregated by treatment with 20 µL 0.1 M HCl.

As stated previously, the concept of the *E. coli* O157 biosensor is based on the SPR band produced by the coherent oscillation of free electrons in colloidal GNP solutions [18]. Upon aggregation of these GNPs, the distance-dependent nature of the SPR band leads to a color change from red to blue. The dextrin-coated GNPs utilized in this study displayed a clear absorbance peak at 520 nm, with shifts to higher peak wavelengths after HCl application (Figure 3). As predicted, application of HCl to the solution disrupted the electrostatic repulsion maintaining the colloid GNP suspension. Thus, the mechanism of action used in other colorimetric biosensor applications was validated. This biosensor utilizes *E. coli* O157 DNA as the target analyte, which anneals to the probe-functionalized GNPs under thermocycler conditions previously described. For this research, it was hypothesized that DNA-GNP conjugates would be protected from aggregation, leading to the output signal of a red solution in target samples. Non-target samples would turn purple/blue due to the lack of GNP protection by target DNA. Therefore, this biosensor can produce a quantifiable signal corresponding to the presence or absence of the target analyte. The signal can be measured visually or using a spectrophotometer.

### 3.2. Optimization and Specificity Testing of Pure E. coli O157 Cultures

Initial optimization of the colorimetric biosensor resulted in the application of 10 µL 0.1 M HCl for all further analyses (Figure S1). Optimized analysis time was determined to be 10 min after HCl application (Table S1). Thus, all analysis steps from sample preparation to colorimetric analysis could be completed in approximately 30 min. This short duration is primarily due to the surface functionalization of the GNPs. As the coated GNPs have carboxylic acid (-COOH) groups, they form non-covalent interactions with the amine-functionalized DNA probes, leading to almost instantaneous GNP-probe functionalization.

**Figure 4:**
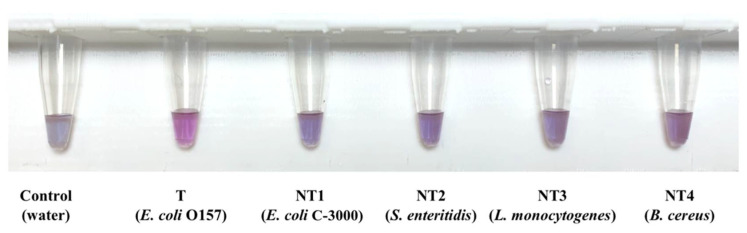
Visual results for one specificity trial using specific *E. coli* O157 biosensor.

After optimization, specificity of the biosensor was analyzed. Each specificity trial included one water control, one target sample (*E. coli* O157), and four non-target species and strains: *E. coli* C-3000 (NT1), *S.* Enteritidis (NT2), *L. monocytogenes* (NT3), and *B. cereus* (NT4). All DNA samples were tested at a concentration of 10 ng/µL. Visual results are displayed in Figure 4.

**Figure 5:**
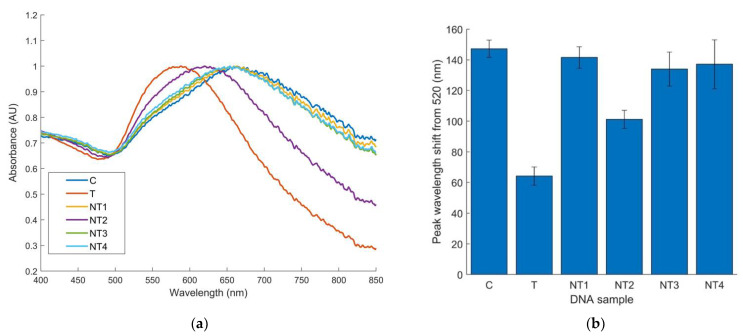
(**a**) Normalized average absorbance spectra for 9 replicates of *E. coli* O157 biosensor; (**b**) *E. coli* O157 specificity results analyzed by peak wavelength shift from 520 nm and conducted with 10 ng/µL of DNA, 9 replicates per sample (*n* = 9). Error bars represent 95% confidence intervals. (C, water; NT1, *E. coli* C-3000; NT2, *S.* Enteritidis; NT3, *Listeria* spp.; NT4, *B. cereus*).

A clear peak shift for control and non-target species is visible on the absorbance spectra when compared to the target samples (Figure 5a). The mean wavelength shift for target samples was 64 nm, while non-target samples showed average shifts from 101–142 nm. Peak wavelength shifts from all nine replicates of each sample type were also utilized for creating 95% confidence intervals. Figure 5b displays average peak shifts from 520 nm and the 95% confidence interval for each sample type. To determine the significance of the biosensor specificity, Kruskal–Wallis and non-parametric Student–Newman–Keuls tests were implemented. The tests found statistically significant differences between the peak wavelength shift of target samples compared to all non-targets and the control with 95% confidence.

Importantly, the results for *E. coli* C-3000 also indicated specificity of this biosensor for target strains within the *E. coli* species. As *E. coli* C-3000 does not contain the target gene (Stx1) or complementary sequence to the probe, the samples with this DNA display GNP aggregation consistent with a negative result. Thus, the biosensor can specifically detect Shiga-toxin-producing *E. coli* strains, which contain the Stx1 virulence gene [41]. This specificity is essential as non-STEC *E. coli* strains that do not cause disease are often found in natural microflora [42].

There was a smaller peak wavelength shift for *S.* Enteritidis compared to other non-target DNA species. While peak wavelength shift ranged from 130–140 nm for other non-targets, the mean peak wavelength shift for *S.* Enteritidis was 101 nm (Figure 5a). The exact cause of this is unknown, because the high specificity of the oligonucleotide probe for STEC annealing to *S.* Enteritidis DNA is highly unlikely. Thus, factors unrelated to probe specificity are most likely contributing to these results. As wavelength shift is dependent upon GNP aggregation, it is possible that lower quality DNA could have interfered with the aggregation process. A260/A230 and A260/A280 ratios were within appropriate ranges, but compromised DNA quality is still possible. Other possibilities for this reduced shift must continue to be explored. Despite the reduced wavelength shift, there was still a significant difference between this non-target and the target DNA with 95% confidence, indicating specificity.

### 3.3. Sensitivity Testing of Pure E. coli O157 Cultures

**Figure 6:**
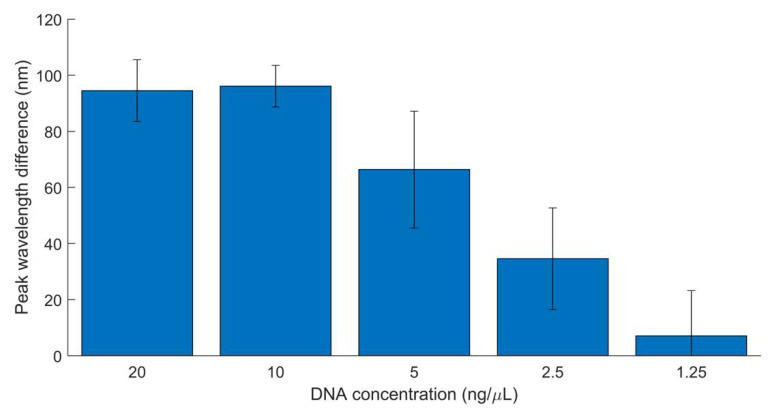
Paired mean difference between target (*E. coli* O157) and non-target (*Listeria* spp.) peak *wavelength*, 20–1.25 ng/µL. Nine replicates. Error bars represent 95% confidence intervals.

To determine the biosensor detection limit for *E. coli* O157, a sensitivity analysis was performed using non-target DNA (in this case, *Listeria* spp.) for comparison. *Listeria monocytogenes* is another dangerous foodborne pathogen, with a 98% hospitalization rate and 16% mortality rate in the United States in 2019 [3]. Both target and non-target DNA were diluted by a factor of two, with testing conducted between 20 and 1.25 ng/µL. Target and non-targets of the same concentration were compared by their mean peak wavelength shifts. Resulting average differences between target and non-target peak wavelengths at each DNA concentration are graphically represented in Figure 6 with 95% confidence intervals.

Results showed a statistically significant difference between target and non-target tubes at concentrations as low as 2.5 ng/µL. Although the lowest tested DNA concentration, 1.25 ng/µL, had a positive mean difference, the confidence interval overlapped zero. Therefore, detection at this concentration is not reliable. This indicates that the biosensor can reliably detect target DNA concentrations at or above 2.5 ng/µL when compared to non-target samples of the same concentration. Because lower concentrations do not produce consistent results, the lowest detection limit of the biosensor is 2.5 ng/µL when a target sample was compared to a *Listeria* spp. non-target sample.

Sensitivity results also indicate that, while the biosensor can detect as high as 20 ng/µL (the highest concentration tested), the linear range of detection is between 2.5 and 10 ng/µL. Above 10 ng/µL, the data appear to show a hook effect, in which a high concentration of a biosensor’s target ligand compared to the capturing molecule leads to decreased or no detection [43,44]. In this case, the DNA concentration (target ligand) most likely oversaturates the probe concentration (capturing molecule), leading to the stagnation of peak wavelength difference shown at 20 ng/µL. If higher concentrations were tested, it is predicted that peak wavelength difference may begin to significantly decrease.

**Figure 7:**
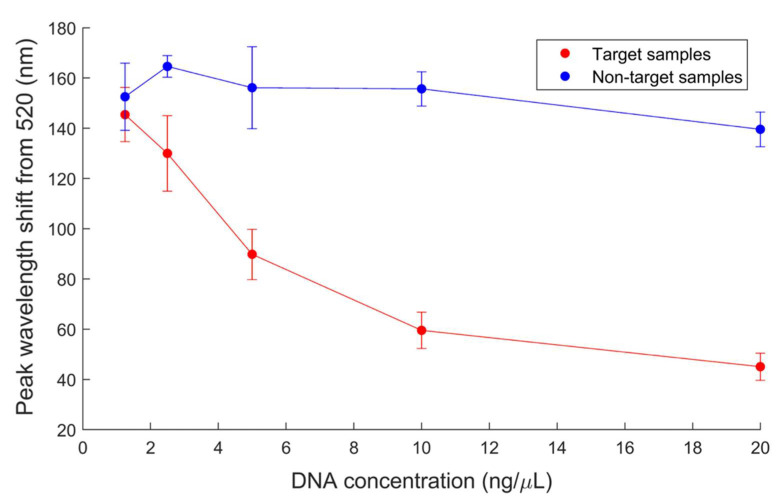
Target (*E. coli* O157) and non-target (*Listeria* spp.) peak wavelength shift from 520 nm at varying concentrations, 1.25–20 ng/µL. Error bars represent 95% confidence intervals of 9 replicates.

Notably, the highest non-target DNA concentration tested (20 ng/µL) had a reduced mean peak shift compared to lower non-target concentrations, although significance of this decrease at the 95% confidence level cannot be established (Figure 7). This trend may be explained by the high concentration of DNA strands in the sample interfering with tube aggregation. Thus, this may also contribute to the linear trend disappearing at concentrations above 10 ng/µL.

### 3.4. Detection of DNA from Flour with E. coli O157 Biosensor

**Figure 8:**
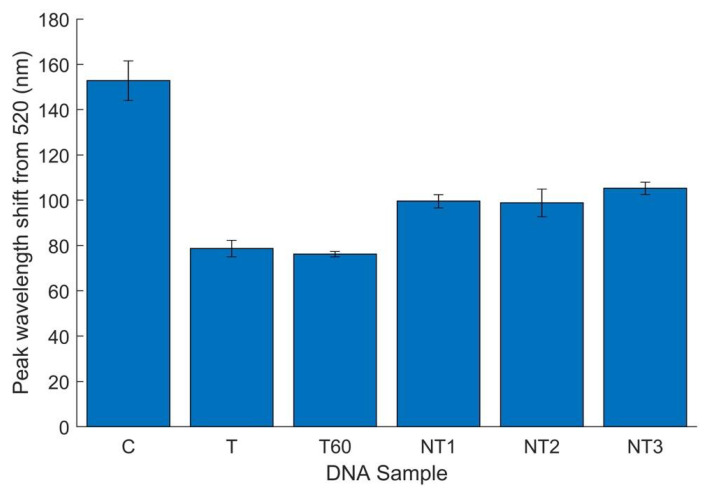
*E. coli* O157 specificity results in flour, analyzed by peak wavelength shift from 520 nm. Six replicates total with DNA concentrations of 60 ± 5 ng/µL unless otherwise noted. Error bars represent 95% confidence intervals. C, water; T, *E. coli* O157 at 83.4 ng/µL; T60, *E. coli* O157 at 60 ng/µL; NT1, *E. coli* C-3000; NT2, *Listeria monocytogenes*; NT3, flour without artificial inoculation.

To establish a proof of concept for this biosensor’s applicability in food matrices, approximately 10^3^ CFU/mL bacteria was extracted from artificially inoculated flour samples using MNPs. After 4 h of growth, DNA was extracted from the sample for implementation in the biosensor. In addition to *E. coli* O157-inoculated flour, DNA was extracted from flour inoculated with other foodborne pathogens (*E. coli* C-3000, NT1, and *L. monocytogenes*, NT2). Thus, testing simulated a situation in which only one bacterial species or strain was present in the food or sample purification steps had been taken. These samples were tested alongside a water control and one DNA sample extracted from magnetically separated flour that had not been artificially contaminated (NT3). DNA extractions from flour not inoculated produced a DNA concentration of 55.6 ng/µL, while the *E. coli* O157-contaminated flour sample produced a concentration of 83.4 ng/µL. Thus, it may be assumed that this difference (approximately 28 ng/µL) is equivalent to the *E. coli* O157 DNA concentration in the target sample. Although variability in DNA yields must be acknowledged, this offers an estimate of the true target DNA concentration. To confirm that positive results for target samples were not due to a higher total DNA concentration, all initial DNA concentrations from flour were tested in the biosensor along with a sample of the *E. coli* O157-containing DNA diluted to 60 ng/µL (T60). Figure 8 displays the mean peak wavelength shift for the six replicates conducted, with error bars representing 95% confidence intervals.

Both target samples (T and T60) exhibited significantly smaller peak shifts than all non-targets, indicating successful detection of *E. coli* O157 from flour with an estimated 28 and 20 ng/µL of target DNA, respectively. With 95% confidence, the Kruskal–Wallis and non-parametric Student–Newman–Keuls tests indicated that each target sample had a significantly smaller peak wavelength shift than all non-target samples (Tables S2–S4). The possible effect of DNA concentration on successful detection was also eliminated, as the T60 sample and all non-targets had similar concentrations. PCR amplification confirmed the presence of the target Stx1 gene in both pure cultures and DNA samples extracted from flour inoculated with *E. coli* O157 (Figure S2). Thus, the biosensor results aligned with PCR analysis.

The samples were then diluted to half their concentration (30 ng/µL total), at which the estimated target DNA concentration was approximately 10 ng/µL. At this concentration, the biosensor was not successful in detecting the target sample. This sensitivity is substantially lower than pure DNA testing (2.5 ng/µL), most likely due to a “dilution effect” in which the presence of additional DNA (e.g., from food matrices or non-target bacteria) in a sample reduces the likelihood of successful detection [27,45]. As flour did not have a high concentration of natural microflora, with only a few colonies noted in MNP concentration experiments, it is likely that the other DNA present is from food matrix components that adhered to the MNPs during extraction. This is further evidenced by the extraction of 55.6 ng/µL of DNA from samples not artificially inoculated (NT3).

### 3.5. Improving Biosensor Sensitivity and Accessibility

Although the lowest detection limit of 2.5 ng/µL was achieved in pure cultures, the biosensor had a higher limit of detection (estimated between 10–20 ng/µL of target DNA) when detecting DNA extracted from flour. There are multiple potential causes for this reduced sensitivity. For one, it is possible that DNA extracted from the food itself interferes with detection. Food particles were clearly visible in most concentrated samples, and the high concentration of DNA from pure flour samples despite the low concentration of natural microflora indicates that some food DNA was most likely extracted. In addition to the aforementioned dilution effect caused by the presence of food DNA, food particulates such as carbohydrates and fats are also known to interfere with DNA-based detection assays [46,47]. This presence of food particles could potentially be addressed through upstream process modifications; for instance, washing the concentrated sample in PBS and repeating magnetic extraction could reduce the food particles present in the sample selected for DNA extraction.

While low-cost materials and simple procedures are already used in this biosensor, accessibility can be further increased through future improvements. For instance, although colorimetric biosensor results were confirmed in this work through absorbance measurements on a NanoDrop, results could also be quantified without the need for spectrophotometry. Smart phone imaging has been shown to be capable of differentiating between aggregated and non-aggregated GNPs by identifying the color change [48], and a phone application could easily be designed for application with this biosensor. At its current state, the other significant equipment need in this biosensor is the thermocycler for DNA/probe hybridization. Due to the simple and static temperature conditions (95 °C for 5 min and 55 °C for 10 min), thermocycler use could be replaced with a water bath. Similar water bath usage has been suggested for isothermal nucleic acid based detection mechanisms [5,49].

## 4. Conclusions

This biosensor offers a rapid alternative to conventional *E. coli* O157 detection assays due to its short experimental duration and limited requirements for analytical equipment. In addition, the visually detectable results allow for eventual elimination of costly analytical equipment currently used for many biosensors [11,12,14,15]. Unlike existing colorimetric biosensors for foodborne pathogen detection [23], this procedure does not require complex functionalization of GNPs before hybridization in a thermocycler, eliminating days of preparation time. As a result, the entire detection assay can be completed in as little as 30 min after genomic DNA extraction. The biosensor can also detect target DNA extracted from large food samples, as well as selectively detect Shiga-toxin-producing *E. coli* strains. Results indicate successful visual and quantitative differentiation between target and non-target DNA from pure cultures at concentrations as low as 2.5 ng/µL. In addition, successful detection of approximately 10^3^ CFU/mL of *E. coli* O157 extracted from flour was achieved with only 4 h of sample growth after bacterial extraction. In future works, upstream bacterial concentration processes can be improved and more inoculated foods can be tested, including foods inoculated with both target and non-target bacterial species simultaneously. With continued improvements, this biosensor can offer cost-effective and rapid detection of foodborne pathogens directly from food matrices.

## Figures and Tables

**Table 1:** Summary of recent biosensor developments for *E. coli* detection.

Biosensor	Analyte, Capturing Molecule	Sample Preparation	Samples	Assay Time	LOD (CFU/mL)	Source
Immuno-fluorescent probes *	Antigens, antibodies	6 h incubation	Water sample	5 min	10^3^	[10]
Impedimetric aptasensor	Outer membrane proteins, aptamer	10 min centrifugation	Pure culture	30 min	10^2^	[11]
SERS *	Antigens, antibodies	N/A	Pure culture	1 h	10^0^	[12]
Smartphone-based fluorescence device	Antigens, antibodies	Suspension in PBS, sandwich immunoassay	Yogurt, Egg	<2 h	1–10	[13]
Fiber optic SPR *	Lipopolysaccharides, antimicrobial peptides	24-h bacterial inactivation	Water, juice	~1 h	5 × 10^2^	[14]
SPR *	Antigens, antibodies	Homogenization and sedimentation (<10 min)	Hamburger, cucumber	<80 min	<50	[15]

LOD: limit of detection; SERS: surface-enhanced Raman scattering; SPR: surface plasmon resonance; * Technique utilizes gold nanoparticles.

## Data Availability

The data presented in this study are available upon request from the corresponding author.

## Supplementary Materials

The following supporting information can be downloaded at: https://www.mdpi.com/article/10.3390/bios12050274/s1, Figure S1: Visual results for optimization of HCl volume; Table S1: Statistical analysis of biosensor results after 5 and 10 min (9 replicates per sample). Readings were stopped after 10 min due to lack of visual differentiation between target and non-target tubes at 15 min; Table S2: Peak wavelength shift data for 6 replicates of *E. coli* O157 GNP biosensor with flour samples and 95% confidence interval testing (NC, water; T, *E. coli* O157 at 83.4 ng/µL; T60, *E. coli* O157 at 60 ng/µL; NT1, *E. coli* C-3000; NT2, *Listeria monocytogenes*); Table S3: Statistical analysis of *E. coli* O157 GNP biosensor with flour samples using Kruskal–Wallis (NC, water; T, *E. coli* O157 at 83.4 ng/µL; T60, *E. coli* O157 at 60 ng/µL; NT1, *E. coli* C-3000; NT2, *Listeria monocytogenes*); Table S4: Statistical analysis of *E. coli* O157 GNP biosensor with flour samples using Non-Parametric Student–Newman–Keuls (NC, water; T, *E. coli* O157 at 83.4 ng/µL; T60, *E. coli* O157 at 60 ng/µL; NT1, *E. coli* C-3000; NT2, *Listeria monocytogenes*); Figure S2: Gel electrophoresis results for PCR-amplified DNA from flour.
