# Supplementary material for: Detection of Unamplified E. coli O157 DNA Extracted from Large Food Samples Using a Gold Nanoparticle Colorimetric Biosensor
Source: Biosensors (Basel). 2022 Apr 26;12(5):274. doi: 10.3390/bios12050274 (PMC9138483; doi:10.3390/bios12050274)
Supplement: Supplementary file 1 [file biosensors-12-00274-s001.zip › biosensors-1690322-supplementary.pdf]

biosensors

MDPI

Supplementary

Detection of Unamplified *E. coli* O157 DNA Extracted from Large Food Samples Using a Gold Nanoparticle Colorimetric Biosensor

Emma Dester [1,2], Kaily Kao [1] and Evangelyn C. Alocilja [1,2,*]

[1] Nano-Biosensors Lab, Department of Biosystems and Agricultural Engineering, Michigan State University, East Lansing, MI 48824, USA; desterem@msu.edu (E.D.); kaokaily@msu.edu (K.K.)
[2] Global Alliance for Rapid Diagnostics, Michigan State University, East Lansing, MI 48824, USA
* Correspondence: alocilja@msu.edu; Tel.: +1-517-432-8672

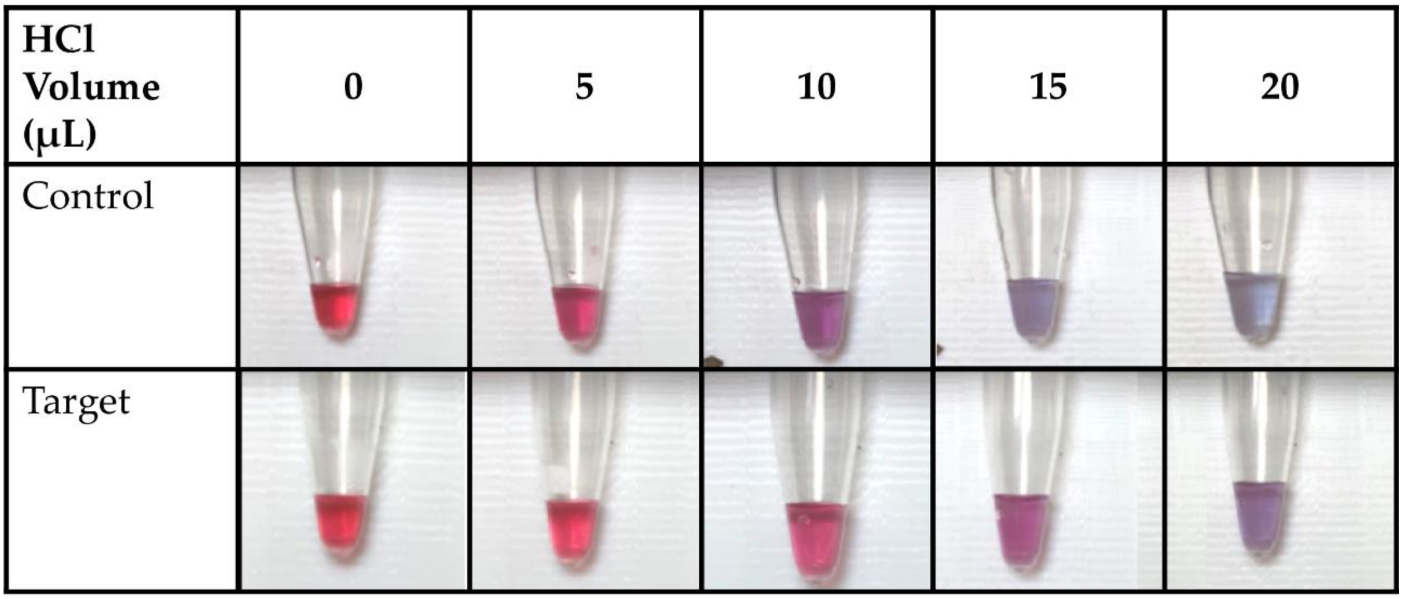

Figure S1. Visual results for optimization of HCl volume.

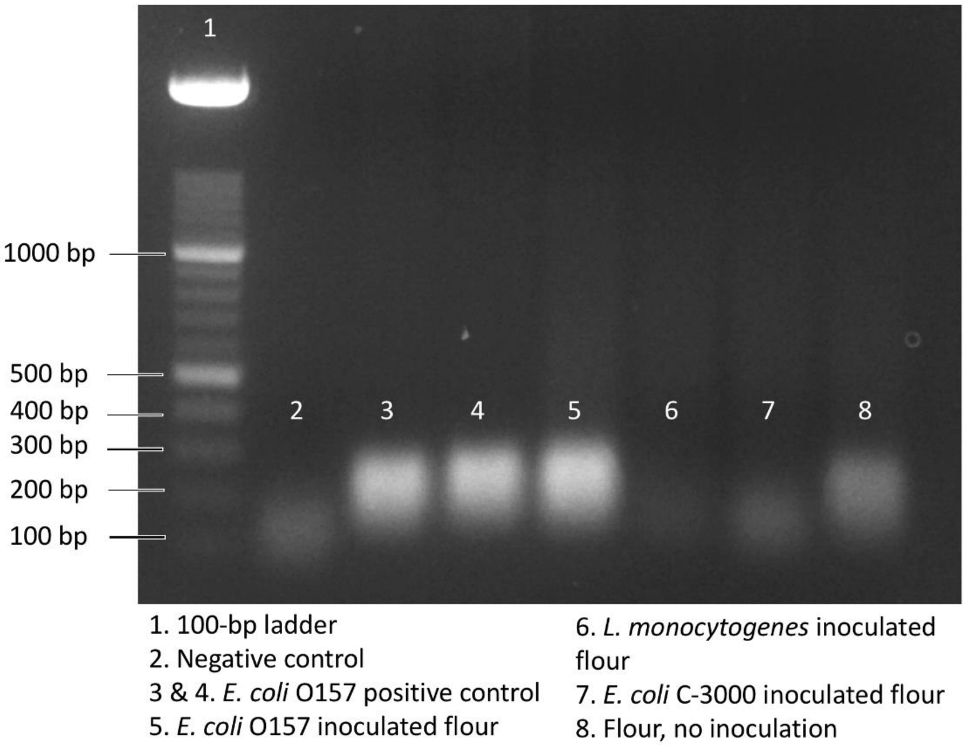

Figure S2. Gel electrophoresis results for PCR-amplified DNA from flour.

Table S1. Statistical analysis of biosensor results after 5 and 10 minutes (9 replicates per sample). Readings were stopped after 10 minutes due to lack of visual differentiation between target and non-target tubes at 15 minutes.

	C	T	NT1	NT2	NT3	NT4
			**Results after 5 minutes**			
**Average**	118.1	54.2	106.8	83.1	103.1	106.9
**SE**	3.73	2.11	4.79	2.39	2.84	5.34
**tcrit**	2.26	2.26	2.26	2.26	2.26	2.26
**95% CI**	109.7 - 126.5	49.4 - 59.0	96.0 - 117.7	77.6 - 88.5	96.6 - 109.5	94.8 - 119.0
			**Results after 10 minutes**			
**Average**	147.2	64.2	141.5	101.2	133.9	137.1
**SE**	2.52	2.62	3.11	2.57	4.90	7.08
**tcrit**	2.26	2.26	2.26	2.26	2.26	2.26
**95% CI**	141.5 - 152.9	58.2 - 70.1	134.5 - 148.5	95.4 - 107.0	122.9 - 145.1	121.0 - 153.1

Table S2. Peak wavelength shift data for 6 replicates of *E. coli* O157 GNP biosensor with flour samples and 95% confidence interval testing (NC = water, T = *E. coli* O157 at 83.4 ng/μL, T60 = *E. coli* O157 at 60 ng/μL, NT1 = *E. coli* C-3000, NT2 = *Listeria monocytogenes*).

	NC	T	T60	NT1	NT2	NT3
**Trial 1**	663.5	597	596.5	618.5	618	628
**Trial 2**	662	606	597.5	618.5	617.5	628.5

**Trial 3**	670.5	597.5	596.5	618.5	610.5	621
**Trial 4**	680.5	597.5	596.5	619	619	624.5
**Trial 5**	681	597	596.5	617.5	629.5	625
**Trial 6**	679.5	597	594	625.5	618.5	625
**Average**	672.83	598.67	596.25	619.58	618.83	625.33
**St. dev.**	8.72	3.60	1.17	2.94	6.10	2.71
**n**	6	6	6	6	6	6
**SE**	3.56	1.47	0.48	1.20	2.49	1.11
**tcrit**	2.45	2.45	2.45	2.45	2.45	2.45
**tcrit*SE**	8.71	3.60	1.17	2.94	6.09	2.71
**UL95%**	681.54	602.26	597.42	622.52	624.92	628.04
**LL95%**	664.13	595.07	595.08	616.65	612.74	622.62

Table S3. Statistical analysis of *E. coli* O157 GNP biosensor with flour samples using Kruskal-Wallis (NC = water, T = *E. coli* O157 at 83.4 ng/μL, T60 = *E. coli* O157 at 60 ng/μL, NT1 = *E. coli* C-3000, NT2 = *Listeria monocytogenes*).

**Ranked data - Kruskal-Wallis**						
	**C**	**T**	**T60**	**NT1**	**NT2**	**NT3**
**Trial 1**	5	30	33.5	18.5	21	9
**Trial 2**	6	25	27	18.5	22.5	8
**Trial 3**	4	27	33.5	18.5	24	14
**Trial 4**	2	27	33.5	15.5	15.5	13
**Trial 5**	1	30	33.5	22.5	7	11.5
**Trial 6**	3	30	36	10	18.5	11.5
**n**	6	6	6	6	6	6
**sum ranks**	21	169	197	103.5	108.5	67
**mean rank**	3.50	28.17	32.83	17.25	18.08	11.17
**Rank**	1	5	6	3	4	2
**Chi^2 calc (H)**	31.32					
**Chi crit**	11.07					

Table S4. Statistical analysis of *E. coli* O157 GNP biosensor with flour samples using Non-Parametric Student-Neumann-Keuls (NC = water, T = *E. coli* O157 at 83.4 ng/μL, T60 = *E. coli* O157 at 60 ng/μL, NT1 = *E. coli* C-3000, NT2 = *Listeria monocytogenes*).

**Initial Calculations**					
**Range**	**2**	**3**	**4**	**5**	**6**
**Pooled SE**	8.83	13.08	17.32	21.56	25.81
**Qcrit**	2.77	3.31	3.63	3.86	4.03
**Critical difference = Qcrit*pooled SE**	24.48	43.34	62.93	83.19	104.00

**Calculation of Individual Differences**			
	**Difference**	**Range**	**Critical difference**
C vs T60	176	6	104.00
C vs T	148	5	83.19
C vs NT2	87.5	4	62.93
C vs NT1	82.5	3	43.34
C vs NT3	46	2	24.48
NT3 vs T60	130	5	83.19

NT3 vs T	102	4	62.93
NT3 vs NT2	41.5	3	43.34
NT3 vs NT1	36.5	2	24.48
NT1 vs T60	93.5	4	62.93
NT1 vs T	65.5	3	43.34
NT1 vs NT2	5	2	24.48
NT2 vs T60	88.5	3	43.34
NT2 vs T	60.5	2	24.48
T vs T60	28	2	24.48
